# Editorial: Circadian rhythm and performance in sports

**DOI:** 10.3389/fspor.2025.1605582

**Published:** 2025-05-07

**Authors:** Georgian Badicu, Felipe J. Aidar

**Affiliations:** ^1^ Department of Physical Education and Special Motricity, Transilvania University of Brasov, Brasov, Romania; ^2^ Graduate Program of Physical Education, Federal University of Sergipe (UFS), São Cristovão, Brazil

**Keywords:** sports, circadian rhythm, sports performance, muscular strength, endurance

**Editorial on the Research Topic**
Circadian rhythm and performance in sports

The circadian rhythm, the biological clock that regulates daily cycles of approximately 24 h in living organisms, has established itself as a factor that would interfere with performance, notably in the sports domain. We feel delighted to have organized the research topic “*Circadian rhythm and sports performance*”, where the advances expressed through the published studies were addressed, with special emphasis on the topic's relevance to athletes, coaches, and sports scientists. This topic, presented, gathered studies that explored circadian processes and their influence on performance, recovery, and decision-making in competitions.

Thus, the objective was to gather evidence that investigated the relationships between biological rhythms and variables related to sports, such as muscular strength, endurance, coordination, and reaction time, among others. The presented studies emphasize the importance of addressing and aligning training and competitions with periods of peak physiological performance and also offer practical strategies to maximize performance and minimize the impacts of circadian misalignments, such as jet lag or atypical competition schedules.

One of the highlights of the supplement was the analysis of circadian variability in key physiological functions. Points such as body temperature, which reaches its peak in the late afternoon, and its relationship with maximum performance in strength and power sports were evaluated, aligning with previous findings on circadian rhythms and physical performance (1). Another study investigated the effects of the sleep-wake cycle on aerobic capacity, observing that endurance athletes could benefit from personalized adjustments to their training schedules. These findings are important in a globalized sports scenario, where international travel challenges biological adaptation.

On the other hand, the influence of the circadian rhythm is not limited to physical performance. In this sense, cognition, essential in sports demanding strategy and precision, also exhibited daily fluctuations. Points such as mental fatigue accumulated throughout the day and the potential impairment it could have on decision-making in team sports were examined. It was also proposed that the timing of mental training sessions could be important, forming part of the training. These findings expanded the scope of sports chronobiology, connecting it to areas such as psychology and neuroscience (2).

Another point of convergence is the discussion about the disruptive effects of misaligned schedules. Nighttime competitions, common in televised events, often place athletes at a physiological disadvantage. It was observed that adaptation strategies, such as controlled light exposure and gradual sleep schedule adjustments, would present themselves as practical solutions to mitigate these negative impacts of competition schedules. These studies are promising for elite and amateur athletes, corroborating studies on chronotherapy (3).

Furthermore, when contextualizing these studies, it is impossible to ignore the broader implications of research on circadian rhythms in sports. In a world where science seeks incremental performance gains, understanding and leveraging biological cycles can be decisive. Complementing this, these insights transcend the sports domain, offering lessons for public health and well-being in societies that operate 24 h a day (4).

The presented studies represent a collective effort to advance our understanding of how biological timing shapes human potential. They promote an advance in chronobiology as a pillar of sports science and inspire new questions: How can this knowledge be integrated into wearable technologies? What role does genetic individuality play in circadian responses? These questions signal a promising future for the field.

We invite readers to explore the works presented here and to consider the circadian rhythm as an ally in enhancing sports performance. We thank the authors and co-editors for their contributions and reiterate our commitment to fostering scientific dialogue on this topic.
